# Constructing a self: The role of self-structure and self-certainty in social anxiety

**DOI:** 10.1016/j.brat.2010.05.028

**Published:** 2010-10

**Authors:** Lusia Stopa, Mike A. Brown, Michelle A. Luke, Colette R. Hirsch

**Affiliations:** a School of Psychology, University of Southampton, Building 44, Highfield, Southampton SO17 1BJ, UK; b Institute of Psychiatry, King’s College, London, UK; c University of Western Australia, Australia

**Keywords:** Social anxiety, Self-concept, Self-concept clarity, Self-structure, Cognitive

## Abstract

Current cognitive models stress the importance of negative self-perceptions in maintaining social anxiety, but focus predominantly on content rather than structure. Two studies examine the role of self-structure (self-organisation, self-complexity, and self-concept clarity) in social anxiety. In study one, self-organisation and self-concept clarity were correlated with social anxiety, and a step-wise multiple regression showed that after controlling for depression and self-esteem, which explained 35% of the variance in social anxiety scores, self-concept clarity uniquely predicted social anxiety and accounted for an additional 7% of the variance in social anxiety scores in an undergraduate sample (*N* = 95) and the interaction between self-concept clarity and compartmentalisation (an aspect of evaluative self-organisation) at step 3 of the multiple regression accounted for a further 3% of the variance in social anxiety scores. In study two, high (*n* = 26) socially anxious participants demonstrated less self-concept clarity than low socially anxious participants (*n* = 26) on both self-report (used in study one) and on computerised measures of self-consistency and confidence in self-related judgments. The high socially anxious group had more compartmentalised self-organisation than the low anxious group, but there were no differences between the two groups on any of the other measures of self-organisation. Self-complexity did not contribute to social anxiety in either study, although this may have been due to the absence of a stressor. Overall, the results suggest that self-structure has a potentially important role in understanding social anxiety and that self-concept clarity and other aspects of self-structure such as compartmentalisation interact with each other and could be potential maintaining factors in social anxiety. Cognitive therapy for social phobia might influence self-structure, and understanding the role of structural variables in maintenance and treatment could eventually help to improve treatment outcome.

Current cognitive models of social phobia emphasise the role of mental representations of the self in maintaining social anxiety (Clark & McManus, 2002; Clark & Wells, 1995; Rapee & Heimberg, 1997), and according to Moscovitch (2009), fear of exposing the self is the ‘core fear’ in social phobia. These claims rest primarily on studies that have focused on the content of the self-concept. This content is formed by negative beliefs and assumptions about the self, and negative images of self-reported by socially phobic and socially anxious individuals (e.g. Hackmann, Surawy, & Clark, 1998; Schulz, Alpers, & Hoffmann, 2008; Stopa & Clark, 1993; Tanner, Stopa, & De Houwer, 2006; Turner, Johnson, Beidel, Heiser, & Lydiard, 2003). Experimental manipulations of positive and negative self-images indicate that they may have causal, as well as maintaining effects, on social anxiety (e.g. Hirsch, Clark, Mathews, & Williams, 2003; Hirsch, Mathews, Clark, Williams, & Morrison, 2006).

Any negative self-representation, according to Showers and Zeigler-Hill (2006) depends both on the *availability* (i.e., content) of self-knowledge and on the *accessibility* of this knowledge. They argue that accessibility is partly determined by the structure of self-knowledge and that structure may moderate the impact of negative self-knowledge on self-esteem and depression. Self-structure refers to the way in which self-knowledge is organised and will be discussed in more detail below.

The accessibility of self-representations is at the centre of Brewin’s (2006) retrieval competition hypothesis. He argues that different self-representations compete and that the effectiveness of cognitive therapy depends not so much on changing negative self-representations, as on making competing positive self-representations more accessible. According to Brewin, access to any given self-representation depends on multiple factors including the individual’s current emotional and physiological state, the available retrieval cues and contexts, autobiographical memories that provide the data bank for competing self-representations, as well as beliefs, attitudes and assumptions about the self. It may also depend on the way in which the information about the self is structured.

If accessibility (i.e. structure) is important in addition to availability (i.e. content), and if the self does indeed play a fundamental role in maintaining social anxiety, then we need to look at the role of self-structure in social anxiety, as well as investigating the contents of self-representations. The aim of this paper is to investigate whether three elements of self-structure, namely, evaluative self-organisation, self-complexity, and self-concept clarity, are related to social anxiety in a non-clinical sample. These three different aspects of self-structure are described below together with comments on their potential role in maintaining social anxiety.

Evaluative self-organisation (Showers, 1992, 2000) refers to the way in which positive and negative self-beliefs are distributed between different aspects of the self. A self-aspect refers to a particular self-defined role (e.g. parent, friend, scientist, engineer) that has a set of attributes associated with it. These attributes can be positive (e.g. kind, loyal, and creative) or negative (e.g. dishonest, selfish, and boring). In Showers’ model there are two different types of evaluative self-organisation: evaluative compartmentalisation and evaluative integration. For individuals with a compartmentalised self-organisation, each self-aspect contains primarily positive or primarily negative information (e.g. a kind, caring, loving son, but a selfish, thoughtless, and indifferent friend), whereas, individuals with an integrated self-organisation have overlapping attributes in their different self-aspects (e.g. a loving, caring, thoughtless and selfish son). Evaluative self-organisation also incorporates the importance of the various different attributes to the individual, so an individual who rates positive attributes as more important than negative attributes is described as having a positively compartmentalised or positive-integrated self-organisation, whereas an individual who rates negative attributes as more important has a negatively compartmentalised or negative-integrated self-organisation.

Self-organisation is not static and can change in response to life events and in the course of therapy (Showers, Limke, & Zeigler-Hill, 2004). Although compartmentalisation confers some benefits (for example, it requires fewer cognitive resources than integration), there are also some costs. Positive compartmentalisation is associated with higher self-esteem and more positive mood than integrated self-organisation. However, negative compartmentalisation is associated with lower self-esteem and more negative mood than negative-integrated self-organisation. When an individual with a positively compartmentalised self-organisation is faced with a stressor, this may trigger the activation of negatively compartmentalised aspects of self, and because these self-aspects do not contain any positive information to buffer them, the individual may be flooded with negative information (semantic and affective information associated with that self-aspect). Showers et al. (2004) argue that, although psychological treatment does not explicitly set out to change self-structure, it may in fact do so. Therapy may facilitate a move from a more compartmentalised to a more integrated self-organisation, in which information of the opposite valence acts as a buffer, thereby reducing extreme reactions (Showers, 1992).

Although most of the research into self-organisation has been done by social psychologists, Taylor, Morley, and Barton (2007) demonstrated that remitted bipolar disorder and recovered depressed groups both had more compartmentalised self-organisation than non-patient controls, and they suggest that increased compartmentalisation may be a general feature of mood disorders. Compartmentalised self-organisation might contribute to the depression that frequently accompanies social phobia, but could it also contribute directly to social anxiety? There is one study that provides indirect support for the idea that it might. In an unselected sample of undergraduate participants, Zeigler-Hill and Showers (2007) found that individuals with compartmentalised self-organisation were more sensitive to lab-based social rejection and had more unstable self-esteem than individuals with integrated self-organisation.

The second structural aspect of the self that we investigate in this paper is Linville’s (1985) model of self-complexity. In this model, high self-complexity is preferable to low self-complexity because it modulates affective reactions. Self-complexity refers to both the number of self-aspects with which individuals define themselves and the degree of overlap between them. More self-aspects and less overlap constitute greater self-complexity. Linville (1987) argues that a stressful life event will activate the self-aspects most relevant to that event. If the individual has a lot of self-aspects, then only a small proportion of the self is affected. Minimal overlap also means that other related self-aspects are less likely to be activated, and thus high complexity has a protective effect. However, there is mixed support for this hypothesis and in a recent meta-analysis, Rafaeli-Mor and Steinberg (2002) found only weak support for Linville’s (1985) claim that high self-complexity is associated with better psychological well-being.

Despite the lack of robust support for the stress-buffering effects of self-complexity, there are other routes through which self-complexity might influence social anxiety. Renaud and McConnell (2002) found that low self-complexity was associated with a bigger rebound effect on a thought suppression task. Post-event processing (PEP) is a common and distressing response to social events among socially phobic individuals (Rachman, Gruter-Andrew, & Shafran, 2000), and attempts to suppress intrusive thoughts during PEP could produce a rebound effect that makes it harder to terminate processing, thus increasing distress. McConnell et al. (2005) demonstrated that greater self-complexity was associated with poorer physical and psychological outcomes, but only for people who believed that they had little control over their various self-aspects. Many socially phobic individuals believe that they are socially inept and cannot create the desired social impression, which leads to anxiety. For an individual with low self-complexity, this self-aspect may be a prominent part of his or her general self-concept and could contribute to a feeling that “this is the way I am”, resulting in a sense of hopelessness. In the current study, we wanted to explore whether high or low self-complexity was associated with higher levels of social anxiety, but given the extant literature, it was not possible to make a clear directional prediction.

The self is a complex multi-dimensional entity and this raises the question of how individuals maintain a coherent sense of self, and brings us to our final structural concept, namely self-concept clarity. Self-concept clarity describes the degree to which “the contents of the self are clearly and confidently defined, internally consistent, and temporally stable” (Campbell, Assanand, & Di Paula, 2003, p. 122). Baumgardner (1990) suggests that a high degree of certainty about one’s self-concept can contribute to a sense of control about future outcomes, which in turn supports a positive and confident view of self. Conversely, uncertainty about the self-concept is associated with low self-esteem, less positive affect towards the self, temporal instability in self-descriptions, and lower congruence between perceptions of current and past behaviours (e.g. Baumgardner, 1990; Campbell, 1990). People with low self-concept clarity are likely to be more vulnerable to the effects of external stimuli. This may help to explain why perceptions about the outcome of social events impact on feelings of self-worth in individuals with social phobia to a greater extent than either anxious or non-patient controls (Gilboa-Schechtman, Franklin, & Foa, 2000).

There is already some evidence that people with social phobia are less certain about themselves. Wilson and Rapee (2006) found that socially phobic participants rated more negative personality traits and fewer positive personality traits as self-descriptive compared to non-patient controls, even after controlling for depression. Socially phobic participants were also less confident about their ratings (both positive and negative) and took longer to make self-relevant decisions on a reaction time task than controls. Wilson and Rapee suggest that lack of certainty about personality traits might result in socially phobic individuals giving undue weight to other people’s opinions (or at least to their beliefs about other people’s opinions), and therefore uncertainty could contribute to the maintenance of the disorder. Moscovitch, Orr, Rowa, Reimer, and Antony (2009) compared socially phobic individuals with healthy controls using a modified version of the Pelham and Swann’s (1989) self-attributes questionnaire, and showed that the patient group rated themselves more negatively than the controls on a range of personality attributes. The controls attributed more certainty and importance to the positive attributes, whereas the socially phobic individuals did not show any difference in certainty and importance ratings between positive and negative traits, suggesting the absence of a positive bias that, if present, could be beneficial in maintaining high levels of self-esteem and confidence in the self.

The two studies described in the present paper build on a sparse literature about the role of self-structure in the maintenance of social anxiety by examining the roles of self-organisation, self-complexity, and self-concept clarity in social anxiety. In the first study, we used a correlational design to examine the associations between social anxiety and the different components of self-structure, and then conducted a regression analysis to assess the contribution of different aspects of self-structure to social anxiety. Social anxiety does not exist in a vacuum and is frequently accompanied by depression and low self-esteem. However, despite the fact that there is shared variance between the three constructs, they can be meaningfully distinguished. For example, Gibb, Coles, and Heimberg (2005) showed that the Liebowitz Social Anxiety Scale (LSAS; Liebowitz, 1987), and the Social Anxiety Interaction and Social Phobia Scales (SIAS and SPS: Mattick & Clarke, 1998) could reliably distinguish social anxiety from depression. As the LSAS is clinician rated and as social interaction anxiety is likely to produce the best range of scores in a non-clinical population, we chose the SIAS to measure social anxiety in our samples.

The low self-esteem associated with both social anxiety and depression reflects the negative view of self that characterises both disorders and is often used to link them (Tennen & Affleck, 1993; Wood & Lockwood, 1999; see Roberts, 2006, for a review). However, social phobia is not limited to negative beliefs about the self, and in one recent study (Kocovski & Endler, 2000), self-esteem was not a direct predictor of social phobia in a multiple regression analysis; the relationship between self-esteem and social anxiety was mediated by fear of negative evaluation. We measured self-esteem and depression in the regression because of the overlap between the three constructs and we wanted to test whether self-structure independently contributes to social anxiety after controlling for them. In the second study, we screened participants with the SIAS and selected high and low social anxiety groups, who were then compared on the various measures of self-structure.

The main hypotheses in the first study were as follows. One, we predicted that negative compartmentalisation, and lower self-concept clarity would be associated with social anxiety. Two, following Showers (1992; Showers & Kling, 1996), we predicted that individuals with a compartmentalised self-organisation who rated positive self-aspects as more important than negative would experience lower levels of social anxiety than those who rated negative self-aspects as most important. Our third prediction was more speculative. We were interested in whether self-organisation and self-concept clarity might interact, and although Campbell et al. (2003) failed to find any correlations between these two measures of self-structure, two recent studies provided some indirect support for this hypothesis. Ayduk, Gyurak, and Luerssen (2009) showed that rejection and interpersonal conflict reduced state self-concept clarity, and Boyce (2009) found that positive integration was associated with higher self-concept clarity and produced less confusion and a less intense emotional reaction when individuals were given the task of integrating new self-knowledge. Accordingly, we tentatively predicted that individuals with more integrated self-organisation and higher self-concept clarity would report less social anxiety than those with more compartmentalised self-organisation and lower levels of self-concept clarity. Finally, we also wanted to explore the role of self-complexity and to see whether high or low complexity contributed to social anxiety.

## Study one: self-structure and social anxiety

### Method

#### Participants

Ninety-eight undergraduates (70 female and 28 male) aged between 18 and 57 (*M* = 21.79, *SD* = 6.82) participated in the study. There were significantly more female than male participants, *χ*^2^ (1, *N* = 98) = 18.00, *p* < .001; however, they did not differ in age (male, *M* = 23.55, [*SD* = 10.07]; female, *M* = 21.14 [*SD* = 5.11]), *t*(96) = 1.59, *p* = .12.

#### Measures

##### Descriptive measures

###### Social Interaction and Anxiety Scale (SIAS; Mattick & Clarke, 1998)

The version of the SIAS that we used contained 19 items that are rated on a five-point scale from 0 (*not at all characteristic or true of me*) to 4 (*extremely characteristic or true of me*). The SIAS is scored by summing the ratings and total scores range from 0 to 76. The internal consistency of the SIAS in this study was high (*α* = .89) and participants had moderate levels of social anxiety overall (*M* = 24.83, *SD* = 11.32).

###### Beck Depression Inventory-Two (BDI-II; Beck, Steer, & Brown, 1996)

The BDI-II consists of 21 items rated on 0 (*not at all*) to 3 (*severely*) scales, and the total scores range from 0 to 63. The BDI-II has excellent psychometric properties (Beck et al., 1996; Dozois, Dobson, & Ahnberg, 1998). In this study, the internal consistency of the BDI-II was good (*α* = .79) and participants had mild levels of depression overall (*M* = 8.78, *SD* = 5.97).

###### Rosenberg Self-Esteem Scale (RSES; Rosenberg, 1989)

The RSES taps global self-esteem and consists of 10 items rated on 1 (*strongly agree*) to 4 (*strongly disagree*) scales. Total scores range from 1 to 40. Positive items were reverse-scored so that higher scores indicated higher self-esteem. The internal consistency of the RSES in this study was high (*α* = .86) and participants reported high self-esteem overall (*M* = 3.10, *SD* = 0.46). The RSES correlates highly with self-concept clarity (*r* = .61; Campbell et al., 1996), which provided another reason to control for the effects of self-esteem when examining the impact of self-concept clarity on social anxiety.

##### Self-descriptive card-sorting task

Self-organisation was measured using Showers’ (1992; Showers & Kling, 1996) version of a card-sorting task originally developed by Zajonc (1960) and adapted by Linville (1985, 1987) to investigate self-complexity. Participants are given a deck of 40 cards, which contains 20 positive and 20 negative personality attribute words. Table 1 shows the words used in this study. The positive and negative words did not differ in length, *t*(48) = 0.83, *p* = .41, number of syllables, *t*(48) = 0.27, *p* = .79, or word-frequency, *t*(48) = 1.81, *p* = .08. Participants sort the cards into groups that describe an “aspect of yourself or your life”. They are instructed to think of as many aspects as they wish that are meaningful in describing their selves. Next, they label each self-aspect (e.g. friend, daughter, student) and record the numbers of the cards that they have selected as descriptive of that self-aspect. There are no limits to how many cards a participant can choose to describe a self-aspect and duplicate cards across self-aspects are permitted. This procedure is repeated until the participant has finished identifying self-aspects.

The card-sorting task provides the following four measures of self-structure:

1. *Self-organisation (integration versus compartmentalisation)*. We followed Showers’ (1992) method of calculating self-organisation, which uses the phi statistic (also known as Cramer’s *V*, Cramer, 1974). Phi is essentially a normalised chi-square statistic in which the expected frequencies represent chance values for the number of positive and negative attributes in each self-aspect (e.g. if the proportion of positive to negative attributes across the whole sort is 6:4 then you would expect the same ratio in each self-aspect), and the observed frequencies are obtained from the actual proportions used in the card sort. Scores can range from 0 (total integration) to 1 (total compartmentalisation). In this sample, overall, participants displayed a slight tendency towards compartmentalisation (*M* = 0.57, *SD* = 0.23).
  Phi scores can only be computed for individuals who have at least two negative attributes in their card sort (Showers & Kevlyn, 1999). Three participants were excluded from the analysis because they did not satisfy this criterion.
2. *Differential importance*. Participants rate the importance of each self-aspect and how positive and negative it is. Differential importance is the within-subject correlation between valence ratings and importance ratings and ranges from −1 (negative attributes are most important) to +1 (positive aspects are most important) (Showers, 1992). Overall, participants rated their positive self-aspects as more important than their negative ones (*M* = 0.64, *SD* = 0.34).
3. *Proportion of negative attributes*. This is calculated by dividing the number of negative attributes in a respondent’s card sort by the total number of attributes, which produces scores that range from 0 to 1. Participants in this sample reported a moderate proportion of negative attributes (*M* = 0.26, *SD* = 0.13).
4. *Self-complexity*. We followed Rafaeli-Mor, Gotlib, and Revelle’s (1999) recommendations and used the number of self-aspects (NSA) and the overlap (OL) between different self-aspects to measure self-complexity. The OL reflects the degree of similarity between the different self-aspects and can range from 0 (no overlap) to 1 [total overlap: see Rafaeli-Mor et al. (1999) for full details of how to calculate the OL]. Rafaeli-Mor et al. demonstrated that these two indices have good reliability, measure self-structure independently of content or valence, and represent two independent dimensions of self-complexity. The mean NSA score was 5.39 (*SD* = 1.43) and the number of self-aspects used by participants in this sample ranged between three and nine. The mean OL score was 0.27 (*SD* = 0.2). The correlation between NSA and OL was *r*(95) = −.17 (*p* = .10), which supports the argument that these two-dimensions of self-complexity are independent. Self-aspects drawn from the social context dominated in frequency, with ‘friend’ (28%), ‘student’ (26%), and ‘romantic partner’ (16%) being identified as the first self-aspect. Other less easily classified terms included examples such as ‘drinker’ and ‘householder’. The specific domains from which participants drew their self-aspect categories mixed both nouns (e.g., friend, student) and evaluative descriptors of the self, such as ‘happy’ or ‘realistic’. Although the majority of participants provided at least one noun self-category, there were three who provided only evaluative descriptors including ‘self-beliefs’, ‘work ethic’, and ‘honesty’ for example.

###### Self-Concept Clarity Scale (SCCS; Campbell et al., 1996)

The SCCS contains 12 items measuring the extent to which an individual’s self-concept is clearly defined and stable. Individuals rate each item on a scale ranging from 1 (*strongly disagree*) to 5 (*strongly agree*). A total score was computed by taking the mean of all the items, with higher scores indicating more self-concept clarity. In this study, the internal consistency of the SCCS was high (*α* = .90) and participants’ mean self-concept clarity score was 3.13 (*SD* = 0.76).

#### Procedure

Participants completed the study in groups of one to seven, seated at individual cubicles. The self-descriptive card-sorting task was completed first, followed by the supplementary ratings for each of the self-aspects in participants’ card sorts, and then by measures of social anxiety, depression, self-esteem, and self-concept clarity, which were counterbalanced across participants to control for order effects. One of the experimenters (MB) remained in the testing room throughout the study.

### Results^1^

#### Correlations between self-structure and other variables

Table 2 shows the correlations between all of the measures included in this study. Social anxiety (SIAS scores) was positively correlated with increased compartmentalisation and with a higher proportion of negative attributes in participants’ card sorts. Social anxiety was negatively correlated with both self-concept clarity and with differential importance (DI), which shows that increases in social anxiety were associated with both reduced clarity and with reduced importance ratings of positive self-aspects. Social anxiety was not significantly correlated with either of the self-complexity measures, but this may have been due to the absence of a stressor and will be addressed in the discussion.

As expected, social anxiety was positively correlated with depression (BDI-II) and negatively correlated with self-esteem (RSES). The BDI-II scores were positively correlated with the proportion of negative attributes and negatively correlated with self-concept clarity, and differential importance. The RSES scores were positively correlated with differential importance and with self-concept clarity, but negatively correlated with proportion of negative attributes. Again, all of these correlations are in the expected direction and are consistent with the literature on self-esteem (Bouvard et al., 1999; Campbell et al., 1996).

#### Multiple regression to investigate the role of self-structure in social anxiety

To investigate the impact of self-structure on social anxiety we conducted a step-wise multiple regression with SIAS scores as the outcome variable. At step 1, we entered BDI-II and RSES scores as predictor variables in order to control for their contributions to social anxiety. At step 2, we entered self-organisation (phi), self-concept clarity, differential importance (DI), and the proportion of negative attributes as predictors. We excluded the two measures of self-complexity (NSA and OL) from the regression because they were not correlated with social anxiety scores. At step 3, we entered the predicted interactions between self-organisation (phi) and differential importance (DI), and between self-organisation (phi) and self-concept clarity. All continuous predictor variables were centred for the purposes of testing interactions (Aiken & West, 1991)

Table 3 summarises the regression analysis. At step 1, the whole model was significant, *F*(2, 94) = 24.33, *p* < .001, *R*^2^ = .35. Depression, *t*(94) = 3.14, *p* < .01, *sr*^2^ = .07, and self-esteem, *t*(94) = −3.77, *p* < .001, partial *sr*^2^ = .1 were both significant predictors of social anxiety. At step 2, the model was significant, *F*(6, 94) = 10.6, *p* < .001, *R*^2^ = .42, but the only unique predictor was self-concept clarity, *t*(94) = −2.50, *p* < .01, partial *sr*^2^ = .04. The inclusion of the self-structure variables at step 2 accounted for an additional 7% of the variance in social anxiety scores.

At step 3, the model was significant,^2^*F*(8, 94) = 8.72, *p* < .001, *R*^2^ = .65, and explained an additional 3% of the variance in social anxiety scores. The interaction between phi and SCC was significant, *t*(94) = −2.06, *p* < .05, partial *sr*^2^ = .03. The interaction between phi and DI was not significant, *t*(94) = 0.34, *p* = .73.

**Fig. 1:**
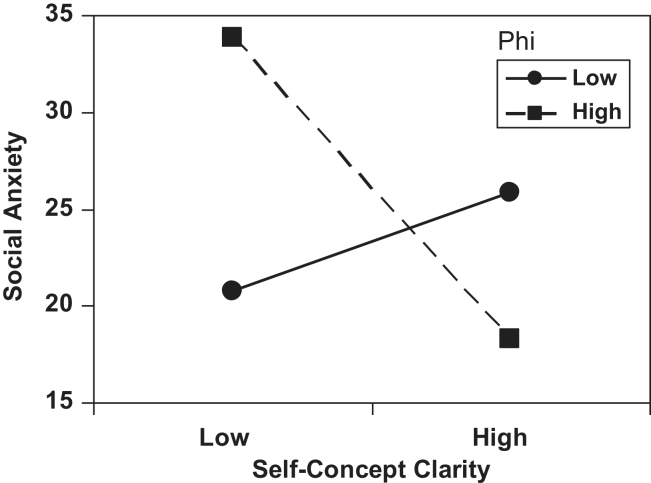
Predicted values for social interaction anxiety, illustrating the interaction of evaluative self-organisation (phi) and self-concept clarity, at values that are one standard deviation below and above the mean for Study 1.

Fig. 1 shows the interaction between compartmentalisation (phi) and self-concept clarity using simple slopes tests that represent scores one standard deviation above and below the mean. Individuals with a compartmentalised self-organisation are more socially anxious when self-concept clarity is low than when it is high (*t*(94) = −4.05, *p* < .001). However, compartmentalised self-organisation produces more social anxiety than integrated organisation when participants have low self-concept clarity (*t*(94) = 4.03, *p* < .001). For individuals with a more integrated self-organisation, level of self-concept clarity did not affect social anxiety (*t*(94) = 1.31, *p* = .20). However, there was a trend for integrated self-organisation to produce higher social anxiety than compartmentalised self-organisation when participants had higher self-concept clarity, *t*(94) = 1.95, *p* = .06.

### Discussion

The first study in this paper examined whether three elements of self-structure – evaluative self-organisation, self-complexity, and self-concept clarity – contributed to social anxiety measured by a scale that can reliably discriminate between social anxiety and depression. The results demonstrate that self-structure does have a role to play in social anxiety even after taking account of the impact of depression and self-esteem, which accounted for 35% of the variance in social anxiety scores when they were entered into the multiple regression as sole predictors. When self-concept clarity and the four indices of self-organisation were added to the regression, they accounted for a further 7% of the variance in social anxiety scores, and the interaction between self-concept clarity and compartmentalisation accounted for a further 3% of the variance in social anxiety scores.

In terms of evaluative self-organisation, we predicted that social anxiety would be associated with more negative compartmentalisation, and although this did appear to be the case when we looked at the correlations, the picture was much more complicated in the regression. Here, none of the measures of self-organisation (phi, differential importance [DI], and proportion of negative attributes) uniquely predicted social anxiety. However, as noted above, compartmentalisation (phi) interacted with self-concept clarity and a compartmentalised self-organisation was associated with higher levels of social anxiety when self-concept clarity was low, but not when it was high; whereas integrated self-organisation was not influenced by self-concept clarity and was preferable to compartmentalised organisation when clarity was low.

In this study, there was no correlation between either of the measures of self-complexity (number of self-aspects and overlap between self-aspects), which seems to indicate that self-complexity does not have a role to play in social anxiety. However, before we reach this conclusion, it is important to note that the absence of an effect could be due to the fact that participants completed the study in conditions of low stress. There is evidence to suggest that self-complexity has an effect when participants are put under stress (Linville, 1985, 1987; Showers, Abramson, & Hogan, 1998). If this is the case, then the methodology of this study would not have allowed us to discover how self-complexity affects social anxiety and it would be premature to conclude that this element of self-structure does not contribute to social anxiety. Subsequent investigations of self-structure should consider measuring self-complexity before and after a socially relevant stress test, such as the Trier Social Stress Test (TSST; Kirschbaum, Pirke, & Hellhammer, 1993).

The results of this study clearly point to self-concept clarity as being one of the important structural variables contributing to social anxiety. Self-concept clarity was the only unique predictor of social anxiety when the structural variables were entered into the regression separately, and there was a significant interaction between self-concept clarity and compartmentalisation. The importance of self-concept clarity in the first study is consistent with both Wilson and Rapee (2006) and Moscovitch et al. (2009), who found reduced certainty about self-variables in participants with social phobia. Showers et al. (2004) suggest that therapy may lead to changes in evaluative self-organisation, but it is also possible that therapy may help individuals to have a clearer and more consistent conception of themselves. However, in the present study, we relied on a single self-report measure of self-concept clarity, which did not take valence into account and could have been subject to social desirability bias. If self-concept clarity does indeed have an important contribution to make to social anxiety, then we need to overcome these problems. To this end, we conducted a second study in which we used a computerised measure of self-consistency as well as the questionnaire measure used in the first study. The computerised task included separate measures of positive and negative consistency, and because it did not rely on self-report, it was much less likely to be subject to social desirability biases.

## Study two: self-concept clarity and social anxiety

### Introduction

In study two, we compared high and low socially anxious participants on the same measures of self-structure that we used in study one, and included a computerised measure of self-concept clarity, which was an adapted version of Markus’ (1977) me/not-me self-description task. This task allowed us to measure consistency of self-view, which is another way of conceptualising self-concept clarity, and also to sample the individual’s degree of confidence about self-related judgments. The me/not-me task also allowed us to derive separate measures of positive and negative consistency; for example, high socially anxious individuals may be quite certain about negative aspects of self-view, but less certain about positive aspects of self-view. By comparison, low socially anxious individuals may be clear and confident about both negative and positive aspects of self-view.

We predicted that the high socially anxious group would have lower self-concept clarity overall and that they would be less consistent and less confident in their judgments about whether words were self-descriptive. We thought it would be likely that high socially anxious participants would be more negatively and less positively consistent than low socially anxious participants and that they would have a more compartmentalised self-organisation. Based on the findings of the first study, we did not expect to find any differences between the two groups in self-complexity.

### Method

#### Participants

Individuals from a local university (students and staff) and from the local community were screened using an on-line version of the SIAS (Mattick & Clarke, 1998). Participants who scored one standard deviation above (29) or below (9) Mattick and Clarke’s undergraduate mean (19.0) were invited to take part. The SIAS was repeated at the time of testing and participants were excluded if their scores did not fall within the cut-offs. The high socially anxious group (5 males, 21 females) had a mean age of 28.59 (*SD* = 13.65) and the low socially anxious group (9 males, 17 females) had a mean age of 27.23 (*SD* = 14.38). There were no significant differences between the two groups in either age, *t*(50) = −0.33, *p* = .75, or in gender composition, *χ*^2^(1, *N* = 52) = 1.56, *p* = .21. Participants were screened for social phobia on the Structured Clinical Interview for DSM-IV-TR Axis 1 Disorders, Non-Patient Edition (SCID-I/NP; First, Spitzer, Gibbon, & Williams, 2002) of the SCID by one of the experimenters (MB) who was trained by LS (an experienced Research Clinical Psychologist). Eight out of 26 (30.8%) high socially anxious participants met DSM-IV criteria for social phobia. None of the low socially anxious participants met criteria for social phobia. All of the SCID interviews were recorded and LS coded 28% of the interviews without knowing the status of the participant. There was 100% agreement between the two coders on the diagnostic status of the participants in the sample used to test the reliability of the coding. Table 4 shows the mean scores for each group for the SIAS, BDI-II, and RSES. These scores were compared using multivariate analysis of variance. There was a significant effect of group, *F*(1, 51) = 66.03, *p* < .001, *η_p_*^2^ = .88. As expected, the high socially anxious group had significantly higher scores on the SIAS, *F*(1, 51) = 268.12, *p* < .001, *η_p_*^2^ = .84, and the BDI-II, *F*(1, 51) = 11.39, *p* < .01, *η_p_*^2^ = .19, and significantly lower scores on the RSES, *F*(1, 51) = 13.84, *p* < .01, *η_p_*^2^ = .22, than the low socially anxious group.

Participants took part in the study for either course credit or a small payment of £6.00 ($10.00).

#### Measures

The SIAS, BDI-II, and RSES are described above. In this study, the internal consistencies of the SIAS, BDI-II, and RSES were all high (*α*s = .96, .94, and .91 respectively). The self-descriptive card-sorting task and the Self-Concept Clarity Scale are described in the method section of study one.

##### Me/not-me self-description task (Markus, 1977)

Participants respond ‘Y’ or ‘N’ to individual words presented in the centre of a computer screen depending on whether the word is self-descriptive or not. The word remains on the screen until the participant responds or until eight seconds have elapsed. An asterisk appears on the screen for one second and then participants rate how confident they feel about the ‘Y’/‘N’ response on a 1 (*not at all confident*) to 7 (*extremely confident*) scale. There were 10 practice trials followed by 50 experimental trials.

The me/not-me task used 25 word pairs (*sociable–unfriendly*, *witty–dull*, *comfortable–awkward*, *significant–insignificant*, *clever–foolish*, *capable–incompetent*, *bold–timid*, *interesting–boring*, *confident–embarrassed*, *successful–failure*, *popular–disliked*, *friendly–disagreeable*, *accepted–ignored*, *appealing–uninspiring*, *self-assured–insecure*, *assertive–inhibited*, *kind–selfish*, *patient–irritable*, *mature–immature*, *intelligent–stupid*, *talented–inept*, *adaptable–inflexible*, *hardworking–lazy*, *outgoing–shy*, *imaginative–uninteresting*), which comprised a mixture of trait descriptors selected from the pool of words used to derive the 40 adjectives in Study 1 because most of them were relevant to social anxiety. Order of presentation was randomised across the 50 individual words. Positive and negative words did not differ significantly in length, *t*(48) = 0.50, *p* = .62, *η_p_*^2^ = .01, number of syllables, *t*(48) = 0.57, *p* = .01, *η_p_*^2^ = .28, or in frequency of usage, *t*(48) = 1.62, *p* = .11, *η_p_*^2^ = .03.

The me/not-me task produces three measures of self-structure: consistency, confidence ratings (1–7), and response times. Consistency is defined as saying yes to one word and no to its antonym or vice versa. Consistent responses are scored as one and inconsistent responses are scored as zero. Therefore scores range from 0 to 25 and higher scores reflect more consistent responses. Total consistency scores do not take valence into account, and therefore we constructed separate scores for positive and negative consistency. Positive consistency involves saying yes to a positive word and no to a negative word; whereas the reverse is true for negative consistency. Scores on each scale range from 0 to 25 with higher scores indicating more positive or negative consistency respectively. For a participant who said yes to every positive word and no to every negative word, the total consistency score would be 25; the positive consistency score would be 25 and the negative consistency score would be 0. Although the sum of the positive and negative consistency scores should be equal to the total consistency score, the proportion of items in the positive and negative consistency scales will vary across participants.

#### Procedure

Participants were tested individually. First, they signed a consent form and completed the BDI-II. Next, they completed either the self-descriptive card-sorting task followed by the me/not-me task, or the reverse as presentation order was counterbalanced across participants. Next, they completed the RSES, SCCS, and the SIAS (also counterbalanced across participants). Finally, the researcher administered the SCID-I/NP to all participants.

### Results

An alpha level of .05 was used for all statistical tests, except when we explored interactions where a Bonferroni corrected alpha was used to control for inflation of the Type I error rate.^3^ We used one-tailed tests to compare the groups on the Self-Concept Clarity Questionnaire and on compartmentalisation (phi), as these were essentially replications of the findings in Study 1. All other tests were two-tailed. In order to control for the associations between social anxiety, depression, and self-esteem, all analyses were initially conducted using depression and self-esteem scores as covariates. We report ANCOVAs where the covariates had a significant effect and ANOVAs where they did not.

#### Self-concept clarity

##### Self-Concept Clarity Scale

Table 4 shows the mean scores for all the self-structure variables reported below. High socially anxious participants reported significantly lower self-concept clarity than low socially anxious participants, *F*(3, 52) = 20.45, *p* < .001, *η_p_*^2^ = .30. Depression also had a significant effect on self-concept clarity, *F*(3, 52) = 18.55, *p* < .001, *η_p_*^2^ = .28; as depression scores increased, self-concept clarity decreased, *r_s_* = −.70, *p* < .001. Self-esteem was not significant (*p* = .1).

##### Me/not-me self-description task

###### Consistency scores

**Fig. 2:**
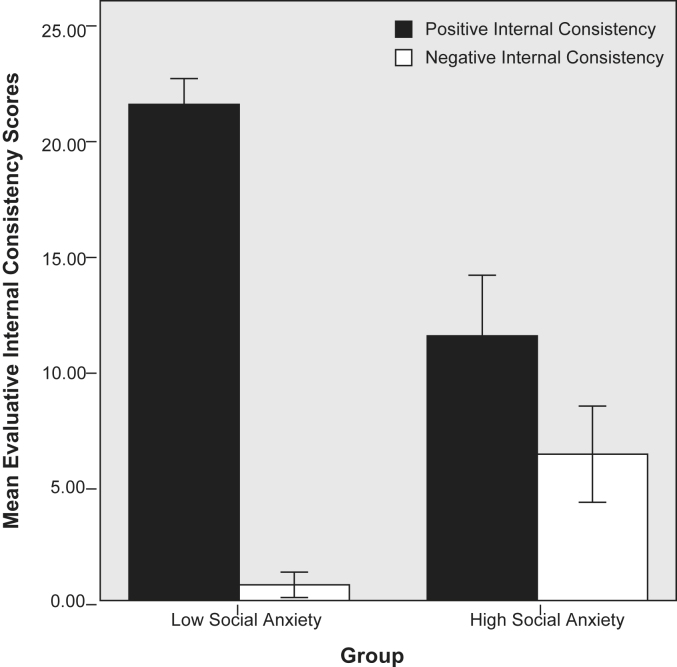
Mean positive and negative internal consistency scores for low and high social anxiety groups for Study 2.

High socially anxious participants had significantly lower total consistency scores than low socially anxious participants, *F*(1, 52) = 33.02, *p* < .001, *η_p_*^2^ = .4, Positive and negative internal consistency were analysed using a 2 (Group) × 2 (Valence) mixed design ANCOVA. There was a main effect of group, *F*(1, 48) = 15.61, *p* < .001, *η_p_*^2^ = .25, but no effect of valence, *F*(1, 48) = 0.15, *p* = .70, *η_p_*^2^ = .00. However, there was a significant group by valence interaction, *F*(1, 48) = 24.17, *p* < .001, *η_p_*^2^ = .34, which is illustrated in Fig. 2. High socially anxious participants gave significantly more negatively consistent responses, *t*(1, 50) = 5.41, *p* < .001, *η_p_*^2^ = .11, and significantly fewer positively consistent responses than low socially anxious participants, *t*(1, 50) = −7.23, *p* < .001, *η_p_*^2^ = .15.

There were no main effects of the covariates (BDI-II, *p* = .7; self-esteem, *p* = .69), but there was an interaction between BDI-II and valence, *F*(1, 48) = 6.9, *p* < .05, *η_p_*^2^ = .13. Increases in depression were associated with increased negative (*r_s_*[52) = .64, *p* < .001) and reduced positive consistency (*r_s_*(52) = −.58, *p* < .001). The interaction between self-esteem and valence just missed being significant, *F*(1, 48) = 4.0, *p* = .052, *η_p_*^2^ = .08.

###### Confidence ratings

Confidence ratings for judgments about the self-descriptiveness of positive and negative trait words were compared using a 2 (Group) × 2 (Valence) mixed design ANOVA. There was a main effect of group, *F*(1, 50) = 7.59, *p* < .01, *η_p_*^2^ = .13, in which high socially anxious participants gave lower confidence ratings to all words. There was no main effect of valence, *F*(1, 50) = 1.30, *p* = .26, *η_p_*^2^ = .03, and no group by valence interaction, *F*(1, 50) = 0.20, *p* = .66, *η_p_*^2^ = .00.

###### Response times

Mean response times to make yes/no decisions were compared using a 2 (Group) × 2 (Valence) mixed design ANOVA. There was a main effect of group, *F*(1, 50) = 3.91, *p* = .05, *η_p_*^2^ = .17, and a main effect of valence, *F*(1, 50) = 7.84, *p* < .01, *η_p_*^2^ = .14, but no valence by group interaction, *F*(1, 50) = 0.21, *p* = .65, *η*_p_^2^ = .00. High socially anxious participants were slower to respond to all words, and all participants took longer to respond to negative compared to positive words.

##### Self-descriptive card-sorting task

The high and low socially anxious groups did not differ on the total number of attributes used in the card-sorting task, *F*(1, 50) = 0.98, *p* = .34, *η_p_*^2^ = .09; or on either of the measures of self-complexity (number of self-aspects, *F*[1, 50) = 0.20, *p* = .65, *η_p_*^2^ = .00; overlap, *F*[1, 50) = 0.21, *p* = .89, *η_p_*^2^ = .00). The high socially anxious participants had a more compartmentalised self-organisation than low socially anxious participants, *F*(1, 50) = 3.46, *p* < .05, *η_p_*^2^ = .00. However, they did not differ on differential importance, *F*(3, 41) = 1.69, *p* = .2, *η_p_*^2^ = .04, or on proportion of negative attributes, *F*(3, 48) = 1.62, *p* = .21, *η_p_*^2^ = .03. Self-esteem had a significant effect on proportion of negative attributes, *F*(3, 51) = 0.7.1, *p* < .05, *η_p_*^2^ = .13, and was on the boundary of significance with differential importance, *F*(3, 44) = 4.07, *p* = .05, *η_p_*^2^ = .09. Increases in self-esteem were associated with a lower proportion of negative attributes, *r_s_*(52) = −.61, *p* < .001. There was a non significant trend for depression to have an effect on the proportion of negative attributes (*p* = .06).

### Discussion

The aim of study two was to extend the findings of Study 1 by comparing self-concept clarity in high and low socially anxious participants using both the questionnaire measure that we had used in Study 1 and the computerised me not-me task. As well as reporting lower levels of self-concept clarity on the questionnaire, the high socially anxious participants had lower overall consistency scores, higher negative consistency and lower positive consistency scores, were less confident in their own judgments, and took longer to make decisions about self-descriptive words on the me not-me task compared to low socially anxious participants. The high socially anxious participants also had a more compartmentalised self-organisation than the low socially anxious group, which is consistent with the correlation between compartmentalisation and social anxiety found in the first study. These findings will be discussed in more detail in the general discussion.

The inclusion of the me not-me task, which is a novel measure of self-consistency that does not rely on self-report and that allowed us to discriminate between positive and negative consistency, is a strength of the second study and contributes to the small evidence base suggesting that uncertainty about the self is characteristic of social anxiety. However, one limitation of this study was our use of analyses of covariance to control for the overlap between social anxiety, depression and self-esteem. Miller and Chapman (2001) argue that analysis of covariance often fails to correct for the variability represented by covariates. Ideally, the study should be replicated with a larger sample using the regression approach that we adopted in Study 1, where depression and self-esteem were entered at step one of the regression analyses, or using a design in which high socially anxious individuals are compared with a depressed group and a low self-esteem group who do not have high levels of social anxiety. Clearly, all of these alternatives have considerable resource implications as they would involve recruiting much larger samples. It is also worth noting that in several of the analyses, the covariates did not have an effect, and therefore these results represented a straightforward comparison between the high and low socially anxious groups.

## General discussion

The results of both studies demonstrate that social anxiety is characterised by reduced clarity or certainty about the self. These effects are present after controlling for self-esteem and depression, which suggests that lack of clarity about the self may be an important contributor to social anxiety. In Study 1, self-concept clarity was the only unique predictor of social anxiety, and also interacted with compartmentalisation in explaining social anxiety scores. Study 2 replicated the finding that high socially anxious participants had lower scores on the Self-Concept Clarity Scale (Campbell et al., 1996), and extended this finding by demonstrating that, although they were also less consistent about the self overall, they were more negatively and less positively consistent about the self than low socially anxious participants. High socially anxious participants were also less confident overall about their judgments than their low anxious counterparts, and this uncertainty may have been reflected by the fact that they took longer overall to make their judgments about the self-descriptiveness of trait words. Clarity or consistency about the self is likely to be related to confidence in judgments about the self, but the modest correlations between these two sets of variable (.35 confidence and SCQ; .4 confidence and consistency scores on the me/not-me task) suggests that clarity/consistency and confidence are two different aspects of self-structure.

The results of these two studies, together with Wilson and Rapee’s (2006) and Moscovitch et al.’s (2009) findings, provide converging evidence for reduced clarity about the self in social anxiety. Paradoxically, high socially anxious individuals were more negatively consistent even though overall they were less consistent in their self-judgments. How could this happen and what effects might it have? Fig. 2 shows clearly that all participants were more positively than negatively consistent, which probably explains the paradoxical effect noted above. This was a non-clinical sample, although some of the high socially anxious participants did meet diagnostic criteria for social phobia, and this might account for the high proportion of positively consistent responses.

The increased negative consistency observed in Study 2 might contribute to the more stable negative perception of self, which is a feature of Hofmann’s (2007) model of social anxiety. A persistent and stable negative self-concept is likely to reduce the individual’s confidence in his or her ability to achieve desired social goals and increase belief in the probability of negative outcomes in social interactions. Once the individual enters a social situation, self-focused attention, negative images of self, safety behaviours and any other strategies that maintain anxiety (see Clark & McManus, 2002, for example) will simply serve to confirm the original negative view of self. At the same time, the emphasis on the importance of positive self-attributes will set up a conflict between the individual’s desired and actual selves and may contribute to overall uncertainty about the self.

Self-concept clarity does in general seem to be beneficial for people and there is a well-documented relationship between high clarity and high self-esteem (Campbell, 1990; Campbell & Lavallee, 1993; Campbell et al., 1996). Baumgardner (1990) points out that self-certainty, which is another way of conceptualising self-concept clarity, promotes positive affect about the self, and she suggests that certainty may contribute to a sense of control that leads individuals to feel that they can influence future outcomes. This is consistent with the suggestion made above. If socially phobic individuals have a more consistent view of negative aspects of self and a less consistent view of positive aspects, then as well as creating more overall uncertainty about the self, they may develop a pessimistic view of future social interaction because they feel that they have little influence or control over the outcomes of these situations.

As well as differences in clarity and consistency, high socially anxious participants also demonstrated less confidence in their judgments about self-attributes. This is consistent with Wilson and Rapee’s (2006) study, where their socially phobic sample had less confidence in ratings made about the self-descriptiveness of personality attributes. Like our sample, their participants also took longer to make judgments about the self. Wilson and Rapee argue that negative evaluation by other people might have more detrimental effects on individuals who are uncertain about themselves. This is an interesting possibility; reduced certainty about the self may lead people to give more weight to other people’s opinions. In the case of social phobia, this might be another route to confirmation of the individual’s negative self-views together with a concomitant difficulty in having any confidence in those positive aspects of self that do exist. There is a consensus across current models of social phobia (Clark & Wells, 1995; Hofmann, 2007; Moscovitch, 2009; Rapee & Heimberg, 1997) that negative self-perception is critical in both the development and maintenance of social anxiety symptoms. The data presented here supports the argument that this emphasis on the self should be supported by examining the individual’s degree of certainty about the self and how any uncertainty might maintain social anxiety.

The relationship between social anxiety and other aspects of self-structure is more complex. The two studies reported here included a number of measures of self-organisation, but the only one which was significant was compartmentalisation. Social anxiety and compartmentalisation were positively correlated in the first study and high socially anxious individuals had more compartmentalised self-organisation than low socially anxious participants in the second. However, compartmentalisation was not a unique predictor of social anxiety in the regression analysis in the first study, but there was a significant interaction between compartmentalisation and self-concept clarity. This interaction was particularly interesting because it suggested that compartmentalised self-organisation is more likely to produce high levels of social anxiety than integrated self-organisation, but only when clarity is low. In fact, integrated self-organisation does not seem to be affected much by different levels of clarity about the self, and as such it might offer some protection against the influence of low clarity on social anxiety. This interaction hints at the complexity of the relationship between the structural elements of self, which in turn will interact with the contents of the self-concept. It would be useful for future research to examine both the contents and the structure of the self-concept and to include clinical as well as analogue samples.

One central aim of current cognitive treatments is to help the individual to develop a more accurate and realistic view of self through the use of methods such as video feedback (Harvey, Clark, Ehlers, & Rapee, 2000), and converging experimental evidence suggests that holding a positive image in mind can reduce social anxiety and improve social performance (e.g., Hirsch, Clark, Mathews, & Williams, 2003). Showers et al. (2004) suggested that a shift to more integrated self-organisation may be one of the beneficial by-products of therapy, but she does not discuss changes in clarity. This idea is consistent with Brewin’s (2006) retrieval competition hypothesis, where he argues that therapy inhibits negative views of the self. What Brewin does not address, however, is what the effect of therapy would be if an individual does not have access to more positive views of self. Butler and Holmes (2009) address this issue indirectly in their discussion about individuals who lack a sense of self in adulthood as a result of abusive childhood experiences. They recommend using imagery and externalising images of the self in order to make connections between thoughts, feelings and memories and to start to develop a representation of the current adult self. It is impossible to know at this stage whether such an approach could also be useful for individuals with social phobia, who lack positive self-representations. Nevertheless, the success of self-imagery manipulations with individuals who experience high levels of social anxiety is encouraging.

Our results suggest that we need to examine both changes in self-organisation *and* any changes in self-concept clarity that occur as a by-product of treatment because there may be important interactions between them. Lack of clarity and/or retaining a strongly compartmentalised self-organisation could represent risk factors for relapse. Of course, at the moment this suggestion is only speculative. However, routine measurement using the Self-Concept Clarity Scale before, during, and after treatment would be one simple way to establish whether self-concept clarity changes and if increased clarity is associated with better maintenance of treatment gains. It could also be useful for clinicians to directly explore the degree to which a patient is uncertain about his or her view of self and to incorporate strategies aimed at increasing clarity and certainty.

The research reported in Study 2 also indicates that it is important to distinguish between clarity about negative and positive aspects of self separately. Although we do not know much about the best way to achieve this end, the use of imagery might be a starting point. The deliberate manipulation and changing of remembered images through techniques, such as imagery rescripting, might help individuals to increase confidence and certainty into a more realistic and functional view of self. Imaginal rehearsal or incorporating a new or more realistic and benign image of self when remembering social interactions are also methods that could be used to help the individual to create a more coherent and consistent view of self. These techniques might work through inhibiting negative and distorted self-views, thus rendering them less accessible. There is also scope for thinking about training consistency of self-judgments, although this would require considerably more research.

Inevitably the research presented in this paper has a number of limitations. First we used a non-clinical analogue sample, although it is noteworthy that a proportion of our high socially anxious group met diagnostic criteria for social phobia. Although this means that the research needs to be replicated on patient groups, the results are consistent with the two previous studies that have used patient samples (Moscovitch et al., 2009; Wilson & Rapee, 2006). We have already discussed some of the problems attendant on using analysis of covariance to control for the shared variance between social anxiety, depression, and self-esteem. Although the regression analysis used in Study 1 to deal with this problem is perhaps a more statistically satisfactory approach, it does require much larger sample sizes and this could be difficult to achieve using patient samples. The design of the studies reported here do not allow us to draw any causal inferences about the role of self-structure in social anxiety, and future studies could address this question by attempting to manipulate self-structure and then observing the effects on social anxiety.

To conclude, the main aim of this paper was to investigate whether self-structure contributed to social anxiety. The two studies reported here suggest that some elements of self-structure are related to social anxiety and deserve further investigation. Self-concept clarity seems to be particularly important, although we have also argued for a role for self-organisation. Self-complexity was not related to social anxiety in either of the two studies reported here, but that may have been due to the absence of any stressor. In practice, of course, structure does not operate independently of content and a more complete understanding of the self will only be achieved when we combine what is already known about the contents of negative self-views in social phobia with this more recent work on how self-structure might contribute to social anxiety.

## Figures and Tables

**Table 1:** Positive and negative personality attributes used in the self-descriptive card-sorting task.

Positive attributes	Negative attributes
Confident	Failure
Friendly	Humiliated
Popular	Inferior
Assertive	Lonely
Successful	Stupid
Articulate	Boring
Appealing	Judged
Charming	Embarrassed
Funny	Vulnerable
Entertaining	Inept
Sociable	Unpopular
Accepted	Idiotic
Interesting	Ignored
Self-assured	Inhibited
Likeable	Ashamed
Pleasant	Shy
Sincere	Unkind
Thoughtful	Insensitive
Realistic	Inflexible
Loyal	Clumsy

**Table 2:** Study 1: correlations between social anxiety, measures of self-structure and depression and self-esteem (*n* = 95).

Measures	SIAS	Phi	PNeg	DI	NASPECTS	OL	SCCS	BDI-II	RSES
SIAS	–	.28**	.40***	−.35***	.14	−.14	−.55***	.50***	−.51***

*Card-sorting task*									
Self-organisation (phi)		–	.40***	−.32**	.26*	−.55***	−.13	.19	−.19
Proportion of negative attributes (PNeg)			–	−.57***	.20*	−.62***	.26*	.40***	−.43***
Differential importance (DI)				–	−.13	.39**	.30**	−.31**	.44***

*Self-complexity*									
NASPECTS					–	−.17	−.15	−.03	−.19
OL						–	.10	−.12	.07
Self-Concept Clarity Scale (SCCS)							–	−.55***	.66***
BDI-II								–	−.51***
RSES									–

*Note*. SIAS, Social Interaction and Anxiety Scale; BDI-II, Beck Depression Inventory-II; RSES, Rosenberg Self-Esteem Scale; NASPECTS, number of self-aspects that participants generated from their card sorts; OL, the similarity between the different self-aspects. *p ≤ .05. **p < .01. ***p < .001.

**Table 3:** Study 1: hierarchical regressions of social anxiety onto measures of self-concept structure and content, depression, self-esteem, and their interactions.

Social anxiety					
Variables	*B*	SE *B*	*β*	*sr*	*sr*^2^
*Step 1*					
Constant	24.83	0.95			
Depression	0.58	0.19	.31	.26	.07***
Self-esteem	−0.87	0.23	−.37	−.32	.10***

*Step 2*					
Constant	24.75	0.92			
Self-concept clarity (SCC)	−0.36	0.14	−.29*	−.20*	.04*
Phi	6.04	4.46	.12	.11	.01
PNeg	9.40	9.29	.11	.08	.00
Differential importance (DI)	−1.38	3.45	−.04	−.03	.00

*Step 3*					
Constant	24.60	0.94			
Phi × SCC	−0.95	0.46	−.18**	−.22**	.03**
Phi × DI	4.23	12.37	−.03	.04	.0007

*Note*: *n* = 95; step 1; *R*^2^ = .35 (*p* < .001, *f*^2^ = 24.33); step 2; *R*^2^ = .42 (*p* < .05, *f*^2^ = 2.79); step 3; *R*^2^ = .45 (*p* < .001, *f*^2^ = 3.19);. PNeg, proportion of negative attributes in participants card sorts; *sr* = semi-partial correlation; *sr*^2^ = squared semi-partial correlation (represents the proportion of variance uniquely accounted for by each predictor, beyond that accounted for by all predictors at that step). *p < .05. **p < .01. ***p < .001.

**Table 4:** Study 2: characteristics of participants in each social anxiety group.

Variable	High social anxiety		Low social anxiety	
	*M*	*SD*	*M*	*SD*
Social Interaction Anxiety Scale	42.19	10.78	6.96	2.02
Beck Depression Inventory-Two	13.92	10.59	5.26	7.66
Rosenberg Self-Esteem Scale	2.76	0.67	3.36	0.46
*Self-Concept Clarity Scale*	*2.53*	*0.82*	*3.82*	*0.80*

*Me/not-me task*				
Consistency				
Total consistency	17.96	3.43	22.38	1.9
Positive internal consistency	11.61	6.58	21.76	2.80
Negative internal consistency	6.42	5.22	0.69	1.37

Confidence ratings				
Positive adjectives	5.07	0.26	5.58	0.56
Negative adjectives	5.01	0.32	5.62	0.58

Reaction times				
Positive adjectives	1892.5	586.1	1602.6	499.8
Negative adjectives	1980.3	528.0	1724.6	436.5

*Self-descriptive card-sorting task*				
Number of self-aspects	5.88	1.97	5.62	2.32
Total number of attributes	64.92	40.22	55.73	27.42
Self-organisation (phi)	0.50	0.23	0.26	0.60
Differential importance	0.37	0.56	0.23	0.59
Proportion of negative attributes	0.33	0.19	0.15	0.16

*Self-complexity*				
Number of self-aspects	5.88	1.97	5.61	2.31
Overlap	0.42	0.21	0.41	0.22

## Footnotes

1 We checked for gender differences on the dependent measures, but there were none (all *p*s < .12) and there were no interactions involving gender. Therefore gender was not considered as a factor in any of the subsequent analyses.

2 For exploratory purposes we also entered all of the two-way interaction at step 3. The overall model was significant, *F*(21, 94) = 6.44, *p* < .001, *R*^2^ = .65, and explained an additional 23% of the variance in social anxiety scores. The following interactions were significant: SCC × phi, *t*(94) = −3.44, *p* < .05, partial *sr*^2^ = .06; SCC × DI, *t*(94) = −3.15, *p* < .005, partial *sr*^2^ = .05; phi × proportion of negative attributes, *t*(94) = −2.19, *p* < .05, partial *sr*^2^ = .02; self-esteem × depression, *t*(94) = 3.34, *p* < .005, partial *sr*^2^ = .05, depression × phi, *t*(94) = −2.19, *p* < .05, partial *sr*^2^ = .02, and depression × proportion of negative attributes, *t*(94) = 2.31, *p* < .05, partial *sr*^2^ = .03.

3 All analyses were initially performed with gender and task order as between-subjects factors. As there were no significant main effects or interactions with either variable, neither is included in the analyses reported below.
